# B-cell hub genes play a cardiovascular pathogenic role of in childhood obesity and Kawasaki disease as revealed by transcriptomics-based analyses

**DOI:** 10.1038/s41598-024-65865-w

**Published:** 2024-07-08

**Authors:** Yuan Chen, Xiaoyi Ji, Yao Ge, Huimin Niu, Xinyi Zhang, Feng Jiang, Chuyan Wu

**Affiliations:** 1 Department of Rehabilitation Medicine, The First Affiliated Hospital of Nanjing Medical University, Nanjing, 210029 China; 2 Department of Pediatrics, Tongren Hospital, Shanghai Jiao Tong University School of Medicine, Shanghai, China; 3 Department of Neonatology, Obstetrics and Gynecology Hospital of Fudan University, Shanghai, 200011 China

**Keywords:** Kawasaki disease, Obesity, Inflammation, Single-cell analysis, Mendelian randomization, Cell biology, Immunology, Biomarkers, Cardiology

## Abstract

The study aims to explore the central genes that Kawasaki disease (KD) and Obesity (OB) may jointly contribute to coronary artery disease. Investigating single-cell datasets (GSE168732 and GSE163830) from a comprehensive gene expression database, we identified characteristic immune cell subpopulations in KD and OB. B cells emerged as the common immune cell characteristic subgroup in both conditions. Subsequently, we analyzed RNA sequencing datasets (GSE18606 and GSE87493) to identify genes associated with B-cell subpopulations in KD and OB. Lastly, a genome-wide association study and Mendelian randomization were conducted to substantiate the causal impact of these core genes on myocardial infarction. Quantitative real-time PCR (qRT-PCR) to validate the expression levels of hub genes in KD and OB. The overlapping characteristic genes of B cell clusters in both KD and OB yielded 70 shared characteristic genes. PPI analysis led to the discovery of eleven key genes that significantly contribute to the crosstalk. Employing receiver operating characteristic analysis, we evaluated the specificity and sensitivity of these core genes and scored them using Cytoscape software. The inverse variance weighting analysis suggested an association between TNFRSF17 and myocardial infarction risk, with an odds ratio of 0.9995 (95% CI = 0.9990–1.0000, p = 0.049). By employing a single-cell combined transcriptome data analysis, we successfully pinpointed central genes associated with both KD and OB. The implications of these findings extend to shedding light on the increased risk of coronary artery disease resulting from the co-occurrence of OB and KD.

## Introduction

Kawasaki disease (KD), also called cutaneous mucocutaneous lymph node syndrome, is an acute inflammatory immune vasculitis primarily observed in children^1,2^. The disease mainly affects small and medium-sized arteries, showing a particular preference for the coronary arteries. This can lead to the development of coronary artery lesions (CAL) in around 25% of untreated patients^3,4^. Patients typically exhibit fever, bulbar conjunctival congestion, oropharyngeal mucosal changes, peripheral limb changes, enlarged cervical lymph nodes, and a polymorphic rash. KD primarily affects children under the age of 5, with boys being at higher risk than girls. The incidence is notably elevated among Northeast Asians, particularly Koreans and Japanese, and varies based on race and season^5^. Despite extensive research, the precise etiology of KD remains elusive. However, its clinical and epidemiological characteristics strongly point towards an infectious origin, implying the participation of immune system activation in the disease's pathogenesis^6^. Among children with KD, CAL stands as the most crucial complication, and in severe instances, it may lead to fatal consequences^4,7^.

Obesity (OB) is widely acknowledged as an independent and modifiable risk factor for numerous chronic diseases, such as hypertension, diabetes, and coronary artery disease, among others^8^. Notably, childhood obesity has emerged as a significant public health concern, with its prevalence continuing to rise in China. The prevalence of obesity and obesity-related diseases is increasing worldwide. Globally, adults with a body mass index > 25 kg/m^2^ increased from 28.8% for men and 29.8% for women in 1980–36.9% for men and 38% for women in 2013^9^. Individuals with OB exhibit a subclinical chronic inflammatory state, which significantly contributes to the development of various metabolic disorders^10^. Research indicates that adipose tissue's immunomodulatory properties, coupled with its association with inflammation and autoimmunity, characterize OB as a low-grade chronic inflammatory state^11^.

KD is characterized by an inflammatory immune vasculitis that predominantly affects the coronary arteries. Childhood-onset autoimmune rheumatic diseases, including KD, face a noteworthy health risk that is modifiable-obesity^12^. Multiple studies suggest that overweight/obesity is an independent risk factor for coronary artery lesions in KD patients^3^. The coexistence of obesity's pro-inflammatory state and KD's inflammatory immune vasculitis leads to a complex pathophysiological link with humoral adipokines and cytokines released from adipose tissue in childhood-onset autoimmune rheumatic diseases, but its complete understanding is still lacking^13,14^.

**Figure 1:**
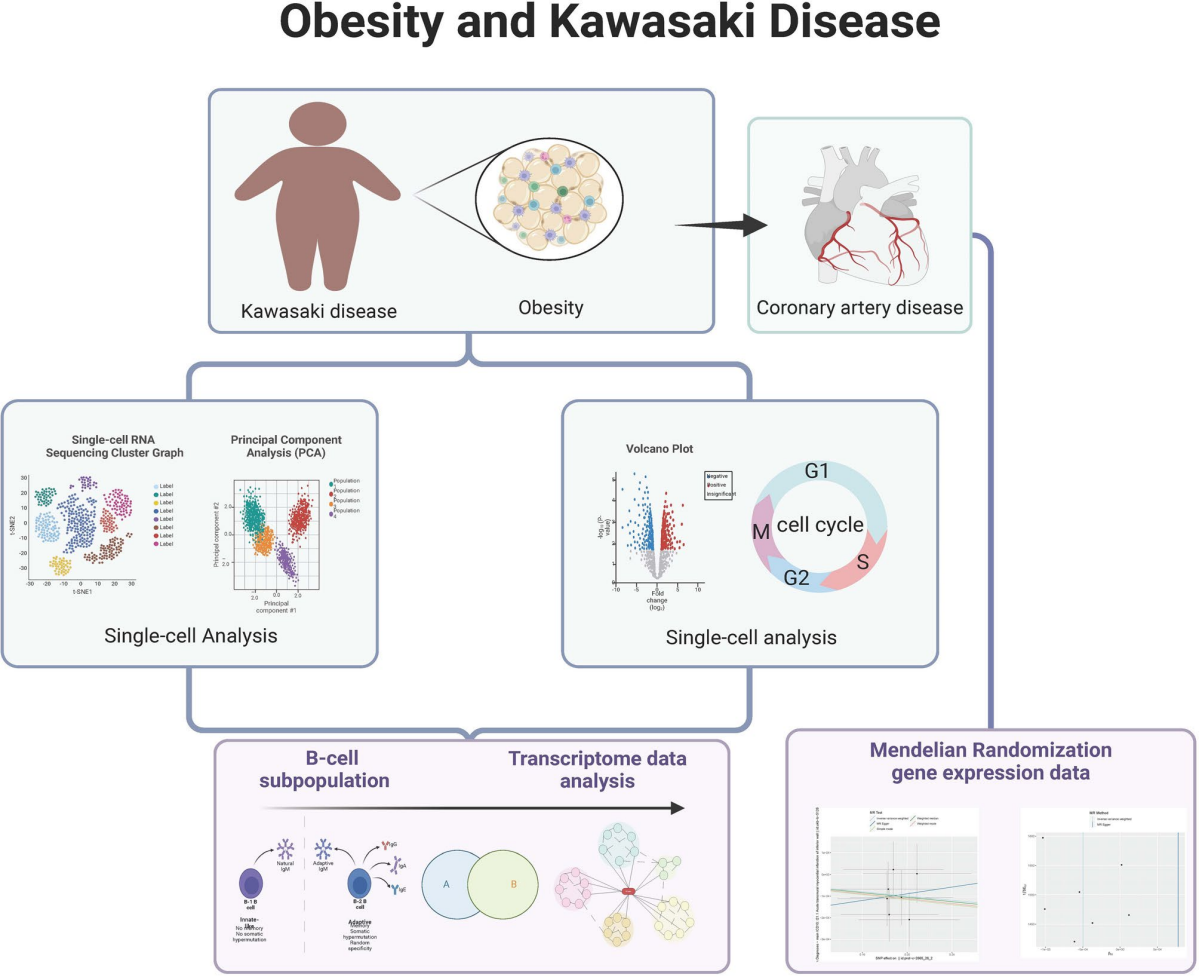
Graphic abstract.

This study delves into the immune microenvironment populations in KD and OB using single-cell analysis to assess their distribution and molecular characteristics. The primary objective is to identify potential shared key genes between KD and OB and explore their interactions and correlations with infiltrating immune cells. The ultimate goal is to gain insights into the underlying pathophysiological mechanisms connecting KD and OB and to determine why the combination of OB with KD worsens complications related to KD-associated cardiovascular diseases (Fig. 1).

## Materials and methods

### Data download

The GEO database (https://www.ncbi.nlm.nih.gov/geo/) provided the single-cell data sets GSE168732 and GSE163830 for KD and obesity. In the KD dataset, there were a total of 6 samples (6 pre-treatment samples), and the OB dataset included 3 samples. Additionally, gene expression profiles for KD and OB were downloaded from the GEO database. The KD gene expression dataset (GSE18606) was based on the GPL6480 platform, comprising 29 samples (20 patients and 9 healthy controls). Moreover, the OB dataset (GSE87493) was derived from the GPL6244 platform, consisting of 32 samples (12 OB children and 20 healthy controls).

### Pre-processing, clustering, and annotation of single-cell sequencing

To ensure consistent analysis, we performed routine data integration of the included datasets using the R package Seurat (version 4.1.4). To eliminate low-quality cells, we employed five Quality Control metrics, necessitating each gene's expression in at least 3 cells, a minimum of 200 genes detected per cell, a cap of ≤ 10,000 genes detected in each cell, and a maximum of ≤ 20% mitochondrial and ribosomal genes. After applying quality control procedures, violin plots were used for visual representation and exploration of correlations between various metrics. To address potential batch effects, we employed Harmony^15^, a method for batch effect removal, and visualized the integrated data using tSNE.

Subsequently, R software was employed to detect highly variable genes, with 2000 genes selected for display on a volcano map, highlighting the top 10. To normalize the data, we employed "LogNormalize" in Seurat and then performed principal component analysis (PCA) to extract the first 20 principal components (PCs). Using these 20 PCs, we established a cell community and clustered the cells using both a graph-based approach (implemented in Seurat v4 of R 4.0.3) and a density-based approach (using the R package dbscan). For visualization, we utilized non-linear dimensional reduction techniques, including tSNE and UMAP, on tissue- and atlas-level datasets. For cell clustering, the "KNN" method was used, and the resolution was set separately. Subsequently, various cell surface markers were utilized for cell annotation^16^.

### Cell cycle analysis and cell–cell communication analysis

To assess cell cycle density, we set the cell cycle position to 0-2P using R software. Subsequently, we analyzed and visualized the proportions of different cell types in various cell cycle stages through tSNE. GSVA analysis of the HALLMARK pathway was then performed for each cell type. To study the molecular interaction networks between cell types associated with KD and OB, we employed CellChat with Single R annotated cell types from both datasets. CellChat^17^, a tool for analyzing intercellular communication networks from scRNA-Seq data, identified ligand-receptor pairs with p-values below 0.05, indicating important interactions between cell subpopulations.

### Immune cell infiltration analysis

The CIBERSORT algorithm, implemented in R software, was utilized to estimate the proportion of the 22 immune cell subtypes. Further analysis selected gene expression matrices with the 22 immune cell subtypes and gene expression profiles of each sample. Subsequently, box line plots were generated to visualize the estimated composition ratios of the 22 immune cell subtypes in each sample, with a p-value less than 0.05 considered statistically significant. We used Gene Set Enrichment Analysis (GSEA), a widely employed method, to assess changes in pathways and biological activities in the expression dataset samples. Finally, to investigate the variations in biological processes among different cell types, GSEA was performed based on gene expression profile datasets from both KD and OB patients^18^.

### Identification of B-cell subpopulation-related genes and pathway enrichments

B-cell subpopulation genes in KD and OB were subjected to Venn diagram analysis, identifying overlapping genes as signature genes for further functional enrichment analysis. We conducted Gene Ontology (GO), Disease Ontology (DO), and Kyoto Encyclopedia of Genes and Genomes (KEGG) pathway analyses^19–21^ using the "cluster profiler" R package^22^ to investigate the biological functions and signaling pathways of differentially expressed signature genes. Significant enriched functions and pathways were identified with corrected p-values < 0.05.

### Protein–protein interaction network analysis and correlation analysis of core genes

The PPI network allowed us to identify key genes and gene modules linked to B-cell subpopulation genesets of KD and OB. The PPI network was constructed using the Search Tool for the Retrieval of Interacting Genes (STRING) database (http://www.stringdb.org/). To rank important genes within the PPI networks, we utilized the Degree algorithm offered by Cytoscape software^23^. Analyzing the overall expression and correlation of core genes involved generating heatmaps and histograms depicting core gene expression levels in both disease and healthy groups using R software. Additionally, we employed the "proc" software tool to create Operating Characteristic curves (ROC curves) for each core gene, enabling us to evaluate the Area Under the Curve (AUC), specificity, and sensitivity of each gene. Moreover, we explored correlations among the 11 core genes and between the core genes and CIBERSORT immune infiltrating cells.

### Quantitative real‑time PCR (qRT‑PCR)

We obtained 38 whole blood samples (six KD, twelve OB, and twenty healthy control samples) from the First Affiliated Hospital of Nanjing Medical University in China between September and December of 2023. The studies involving human participants were reviewed and approved by the Ethics Committee of the First Affiliated Hospital with Nanjing Medical University (2023-SR-563). Patient samples ranged in age from 8 months to 6 years. For white blood cell (WBC) enrichment, we took 3–5 mL whole blood samples from each participant and placed them in tubes containing EDTA. Leukocytes from children with KD, OB, and healthy controls were treated with total RNA extraction, and before receiving aspirin and intravenous immunoglobulin (IVIG), blood samples were obtained from KD children. The Servicebio^®^ RT First Strand cDNA Synthesis Kit (Servicebio, Wuhan, China) was used to execute reverse transcription. The 2× SYBR Green qPCR Master Mix (None ROX) (Servicebio, Wuhan, China) was used for quantitative PCR (qPCR) following the manufacturer's instructions. The thermocycling settings were as follows: thirty seconds of initial activation at 95 °C, forty cycles of fifteen seconds at 95 °C, thirty seconds at 60 °C, and thirty seconds at 60 °C. The internal reference for data normalization was GAPDH. The 2^−ΔΔCt^ approach was used to compute the relative expression. Table S1 contains a list of primers^24^.

### Mendelian randomization

The data used in this study were drawn from open databases. Employing two-sample Mendelian randomization (MR), we aimed to explore the potential causal association between the hub gene, TNFRSF17 (BCMA), and cardiovascular disease risk, specifically focusing on myocardial infarction. For this investigation, we used single nucleotide polymorphisms (SNPs) as instrumental variables (IVs), with the hub gene data obtained from publicly available Genome-Wide Association Study (GWAS) data. For the Mendelian randomization analysis, we focused on TNFRSF17 as the critical gene and myocardial infarction as the representative cardiovascular disease. To access the data on TNFRSF17, visit https://gwas.mrcieu.ac.uk/datasets/prot-c-2665_26_2/, and for myocardial infarction data, go to https://gwas.mrcieu.ac.uk/datasets/ukb-b-5126/. To perform the MR analysis, we used the "TwoSampleMR" Package and implemented inverse variance weighting (IVW) to assess the relationship between hub gene levels and the risk of myocardial infarction. Furthermore, we conducted MR-Egger as an additional sensitivity analysis^25,26^.

### Ethics approval and consent to participate

The studies involving human participants were reviewed and approved by the Ethics Committee of the First Affiliated Hospital with Nanjing Medical University (2023-SR-563). All methods were carried out in accordance with the principles laid down in the Declaration of Helsinki. Informed consent was obtained from all subjects and/or their legal guardian(s).

## Results

### Single cell sequencing data analysis

Following quality control of the single-cell data (Figs. S1 and S2), high variable genes were identified in KD using R software, and the top 10 highly variable genes were presented on the volcano map (Fig. S3a). Utilizing Jackstraw plot (Fig. S3b) and elbow plot (Fig. S3c) analyses, the most significant principal components (top 13 PCs) were selected. For each PC among the top 20, the heatmap displays the top 8 genes (Fig. S3d). Subsequently, the top 13 PCs were employed for UMAP and tSNE analysis to cluster the cells, using a resolution of 2 (Fig. S3g), resulting in 27 annotated cell clusters (Fig. S3e,f). We constructed heatmaps based on differential genes for each cell cluster (Fig. S3h). In KD, the cell annotation heatmap delineated 27 distinct cell subgroups, with the highest score marked in yellow (Fig. S4a). Manual annotation allowed us to classify the 27 cell clusters (Fig. S4b). For improved categorization, we condensed the 27 cell clusters into 10 cell types: Monocyte, NK cell, CD4+ memory cells, Neutrophils, HSC G-CSF, B cell, Platelets, CD8+ T cells, BM, and Myelocyte. Visual representation of these annotated results was achieved using tSNE and UMAP (Fig. S4c,d). In the context of obesity, single-cell data underwent similar analysis and processing (Figs. S5 and S6).

### Cell cycle and functional analysis

**Figure 2:**
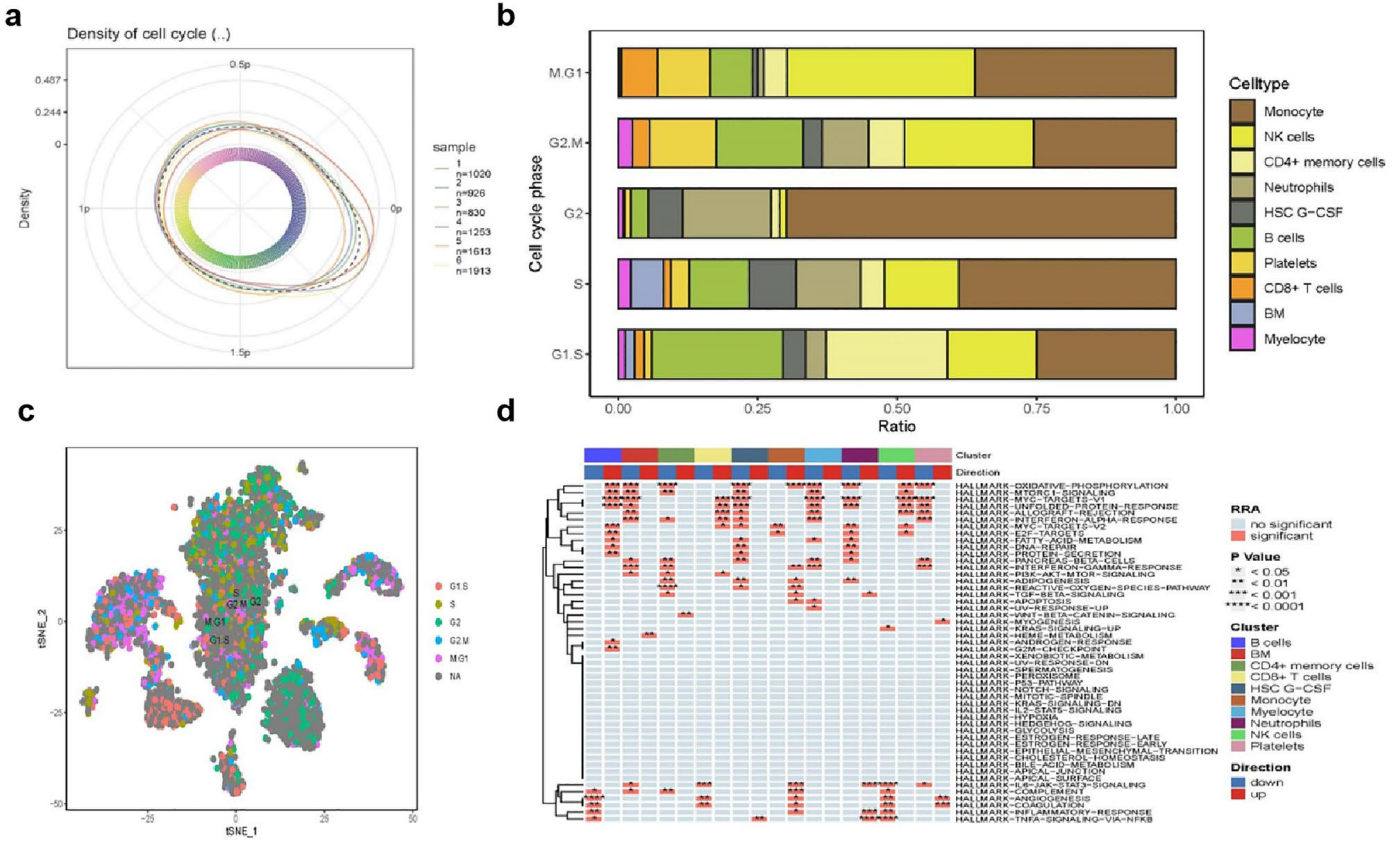
In KD (**a**) Density of the cell cycle. (**b**) Distribution of 10 cell types with different cell cycles. (**c**) Visualizing the cell cycle for tSNE. (**d**) GSVA results of the HALLMARK pathway for different cell types.

Cell cycle position, set at 0-2P, effectively demonstrated cell cycle density in both KD and OB samples (Figs. 2a and S7a). The categorized cell cycle phases (M.G1, G2.M, G2, S, G1.S) showed distinct distribution patterns across different cell types (Figs. 2b and S7b). Utilizing tSNE plots, we further visualized the cell types within each cell cycle phase (Figs. 2c and S7c). GSVA analysis was conducted for the HALLMARK pathway, providing valuable insights into the biological processes of each cell type (Figs. 2d and S7d). In KD, B cells exhibited significant upregulation in the MYC-TARGETS-V1, UPR, OXIDATIVE-PHOSPHORYLATION, and MYC-TARGETS-V2 pathways, while monocytes showed upregulation in OXIDATIVE-PHOSPHORYLATION, IL6-JSS, and COMPLEMENT pathways. In OB, fibroblasts displayed predominant upregulation in E2F-TARGETS, G2M-CHECKPOINT, ROS, and MYC-TARGETS-V1 pathways, while smooth muscle cells showed upregulation in EMT, ANGIOGENESIS, and ROS pathways.

### Cell–cell crosstalk network

**Figure 3:**
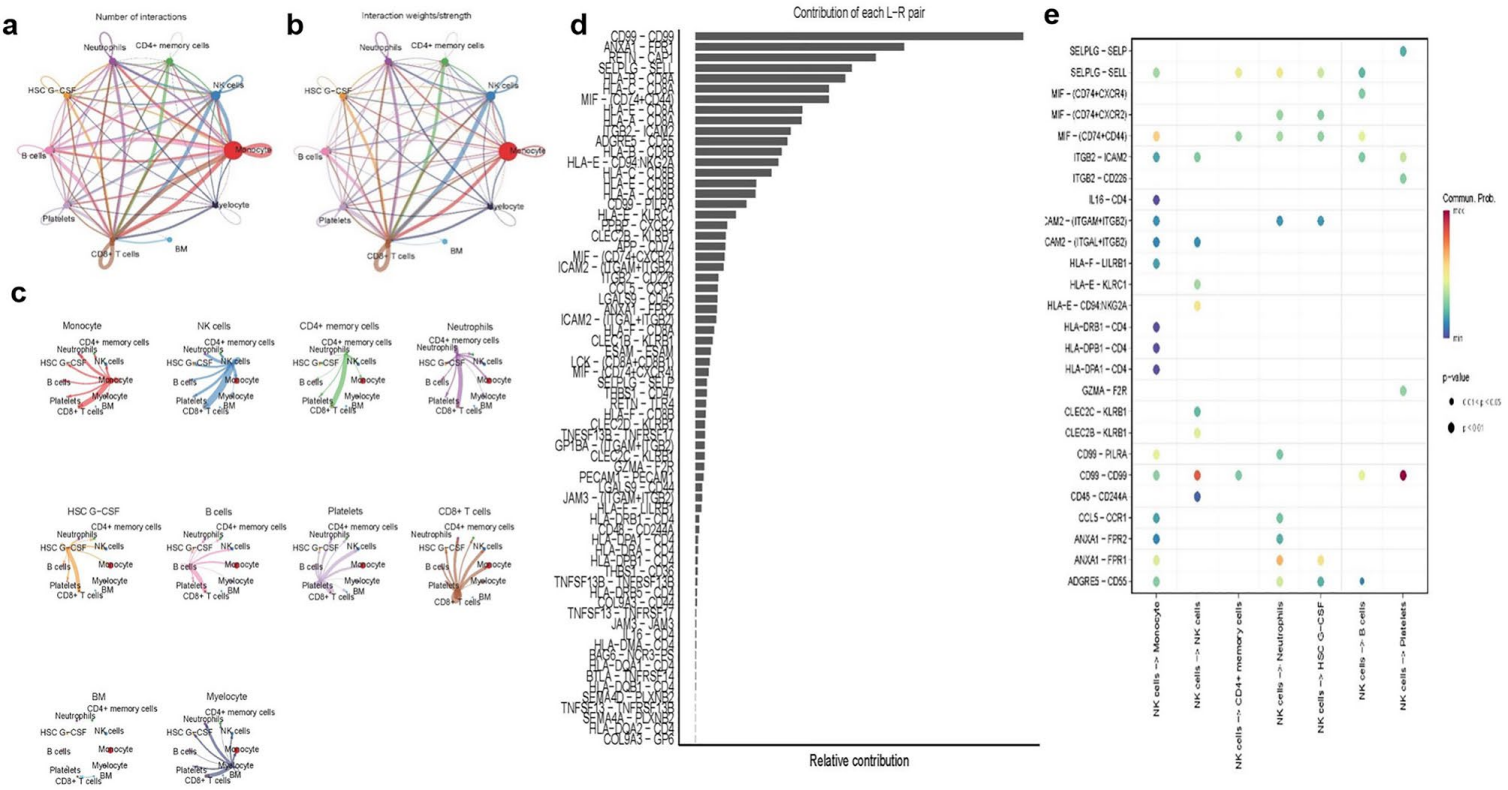
In KD (**a** and **b**) Circular plots of the cellular interaction number (**a**) and interaction strength (**b**) between different cell types. The red line represents an increase in cell–cell contacts, whereas the blue line represents a reduction. (**c**) The circle diagram shows the distinct changes in cell communication for the 10 cell types. The various cell types are shown by the colorful dots. The strength of cellular connections is represented by the thickness of the lines; the thicker the lines, the more powerful the interactions. (**d**) Visualization of ligand-receptor pairs (**e**) Probability of ligand-receptor pair-mediated communication between NK cells and other cell types to identify signals.

**Figure 4:**
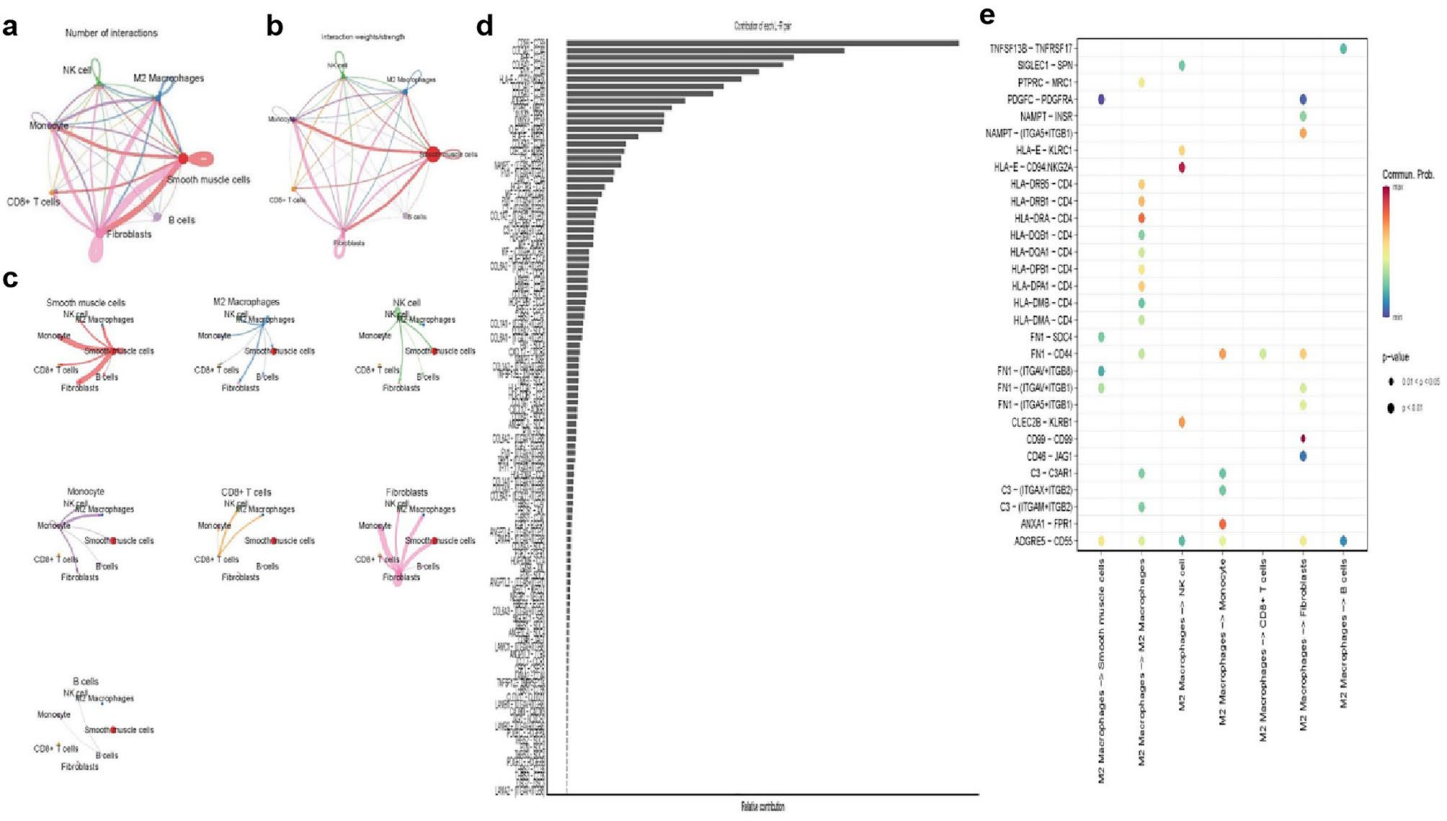
In OB (**a** and **b**) Circular plots of the cellular interaction number (**a**) and interaction strength (**b**) between different cell types. The red line shows an increase in cell contacts, whereas the blue line shows a reduction in those connections. (**c**) The circle diagram shows the distinct changes in cell communication for the 7 cell types. Different sorts of cells are shown by the colored dots. The strength of cellular connections is represented by the thickness of the lines; the stronger the interactions, the thicker the lines. (**d**) Visualization of ligand-receptor pairs. (**e**) Probability of ligand-receptor pair-mediated communication between M2 macrophages cells and other cell types to identify signals.

To elucidate the underlying intercellular communication and cell state transitions in KD and OB, we used CellChat to quantify and visualize the overall changes in cellular communication between different cell types. The results showed that the number of monocyte interactions and the weight/intensity of interactions increased in KD and decreased in NK cells, while the number of smooth muscle cell interactions and the weight/intensity of interactions increased in OB and decreased in M2 macrophages (Figs. 3a-b and 4a-b). The intensity of communication between each cell and other cells is shown in the figure (Figs. 3c and 4c). Many significant ligand-receptor pairs were detected in different cell types, including CD99-CD99 with high expression in both KD and OB. Furthermore, by comparing the communication probabilities between KD and OB, ligand-receptor pairs were identified in KD that were altered from NK cells to other cell types, and in OB that were altered from M2 macrophages to other cell types (Figs. 3e and 4e). The same suggested high expression of CD99-CD99 in KD and OB(Figs. 3d and 4d).

### Identification of disease immune infiltrate cells

**Figure 5:**
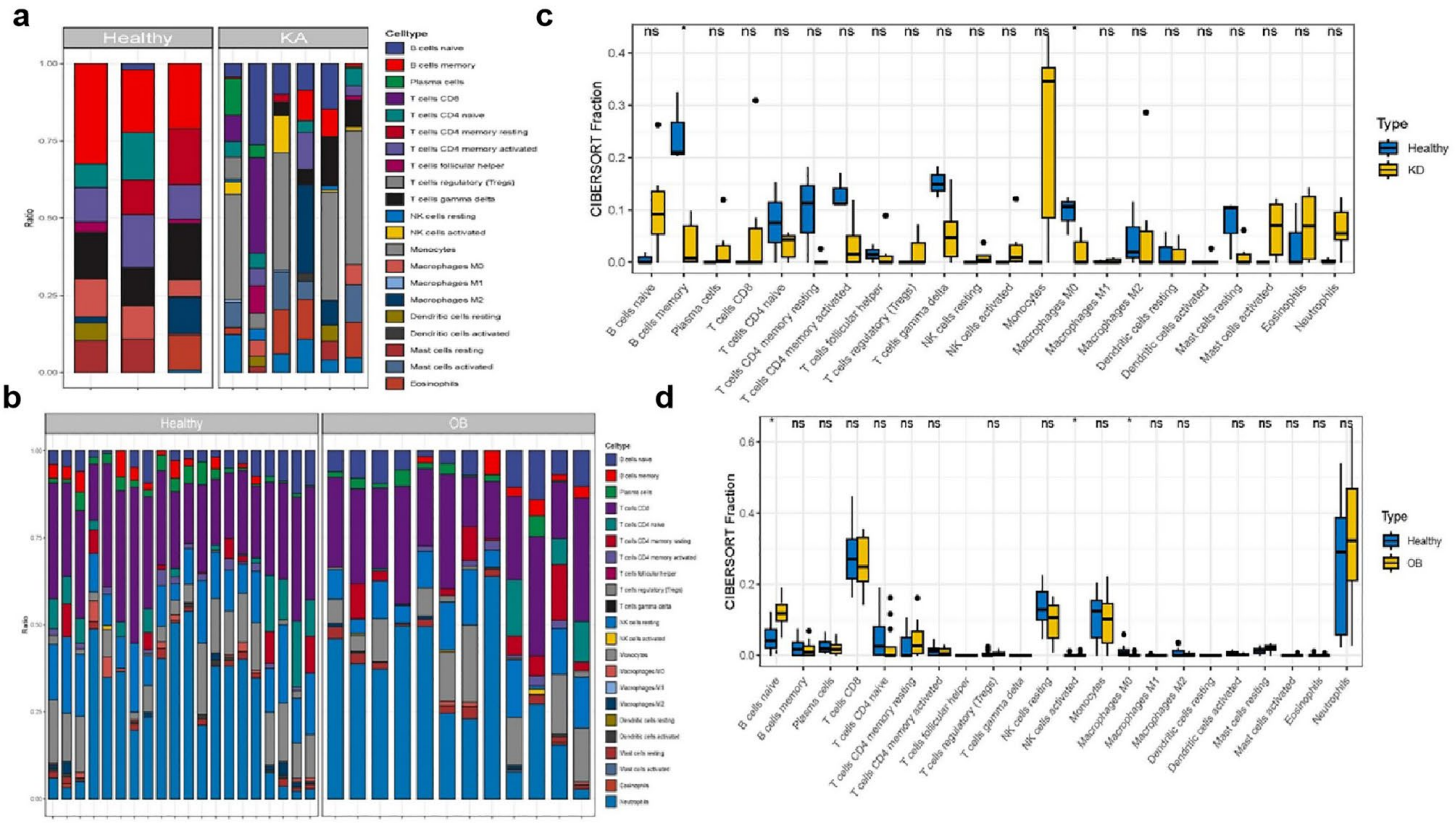
(**a** and **b**) Box-line graphs showing the distribution of 22 immune cell subtypes in healthy and sick groups; various colors denote distinct immune cells. (**c**) Differential expression of 22 immune cell subtypes in the healthy and KA groups,*P < 0.05. The vertical axis represents the cibersort proportion, while the horizontal axis represents the 22 immune cells. (**d**) Differential expression of 22 immune cell subtypes in the healthy and OB groups,*P < 0.05. The vertical axis shows the proportion of the cibersort, while the horizontal axis represents the 22 immune cells.

Gene expression matrices for KD and OB, including 22 immune cell subtypes, were meticulously selected and then normalized for subsequent analysis (Fig. S8). Using the CIBERSORT algorithm in R software, we precisely estimated the proportions of the 22 immune cell subtypes in both healthy and disease groups, and the results were visually represented via box-line plots (Fig. 5a,b). Remarkably, B memory cells and M0 macrophages exhibited higher expression levels in healthy individuals than in KD patients (Fig. 5c). On the contrary, B cells naive demonstrated higher expression in OB, while M0 macrophages were more highly expressed in the healthy group (Fig. 5d).

**Figure 6:**
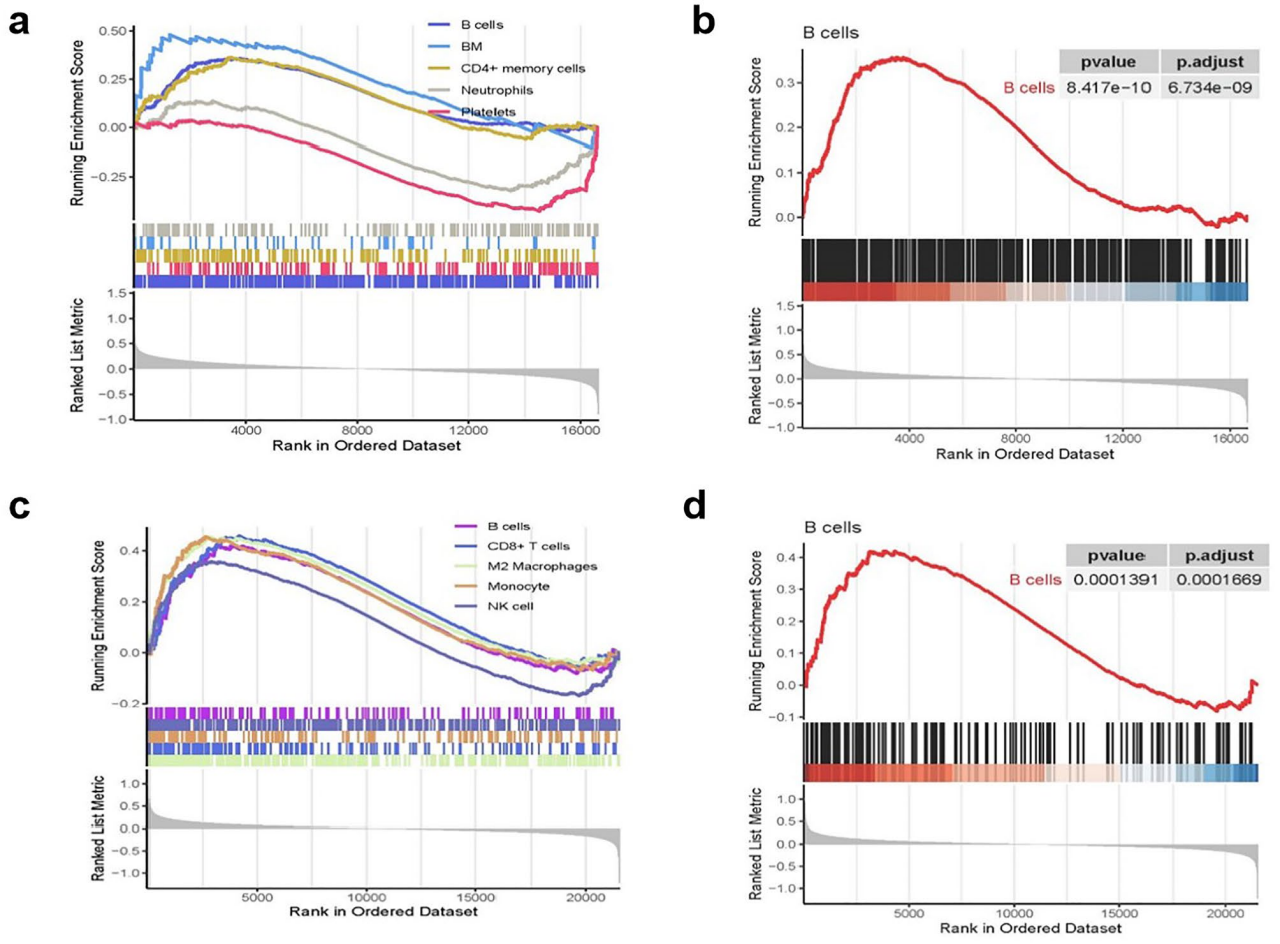
(**a**) Gene set enrichment analysis in KD. (**b**) Gene set enrichment analysis of B-cell characteristic geneset in KD. (**c**) Gene set enrichment analysis in OB. (**d**) Gene set enrichment analysis of B-cell characteristic geneset in OB.

To delve into the intricate biological differences between cells, we conducted a gene set enrichment analysis (GSEA) using the gene expression profiles of KD and OB patients. GSEA, a widely recognized approach, facilitated our assessment of changes in pathways and biological activities in the expression datasets. Noteworthy findings revealed that in both KD and OB, B-cell characteristic genesets were distinctly upregulated in the disease group (Fig. 6a and c). Strikingly, the B-cell characteristic geneset displayed the most robust positive enrichment in KD (p = 8.417e-10) (Fig. 6b) and OB (p = 0.0001391) (Fig. 6d).

### Find shared genes and GO, DO, and KEGG enrichment analyses

**Figure 7:**
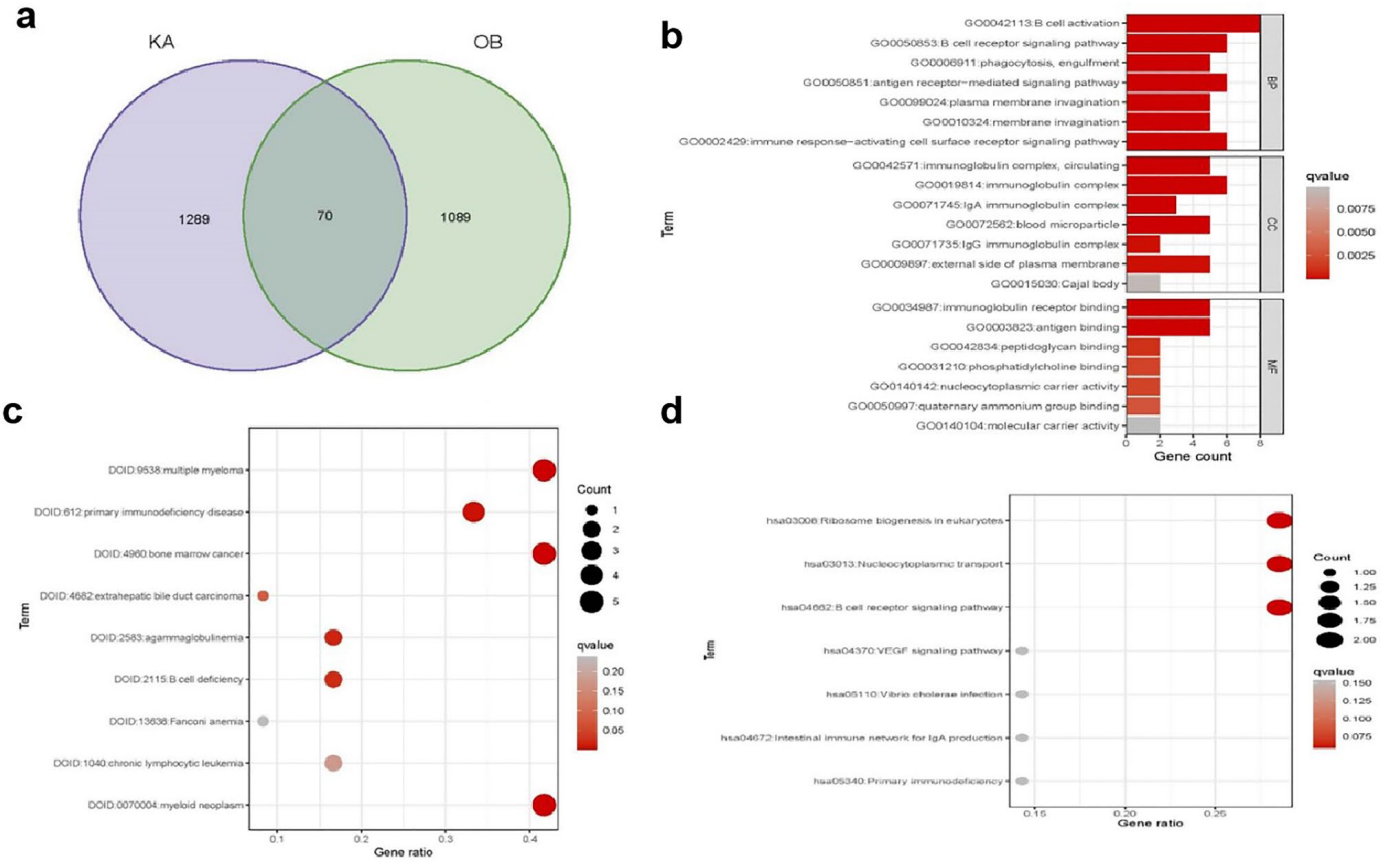
(**a**) We created a Venn diagram to show the shared genes between the differentially expressed genes in the KD and OB B-cell subpopulations. (**b**) GO enrichment analysis. GO enrichment results are depicted in a bar graph with the z-score on the x-axis and the log 10 (adj P) values on the y-axis. (**c**) Create a bubble chart using DO enrichment results. The y-axis represents the log 10 (adj P) value, while the x-axis displays the z-score. A bubble is used to symbolize an illness, and the size of the bubble represents the number of genes in the pathway. (**d**) Results of KEGG pathway enrichment shown in a bubble diagram. The log 10 (adj P) value is depicted on the y-axis, while the z-score is displayed on the x-axis. A bubble that represents a KEGG pathway shows how many genes are involved in the pathway based on its size.

Through the intersection of B-cell subpopulation characteristic genes in KD and OB, we identified 70 shared genes (Fig. 7a). The GO enrichment analysis provided meaningful insights across three categories (qvalue-adjusted), with the top 7 items depicted in Fig. 7b. These crucial biological processes included B cell activation, B cell receptor signaling pathway, antigen receptor-mediated signaling pathway, and immune response-activating cell surface receptor signaling pathway. Furthermore, the cellular component analysis unveiled the involvement of the immunoglobulin complex, circulating, immunoglobulin complex, blood microparticle, and external side of the plasma membrane. On the molecular functionalities front, immunoglobulin receptor binding and antigen binding played significant roles. As for DO enrichment analysis, the DEG concentration was prominently found in multiple myeloma, primary immunodeficiency disease, bone marrow cancer, and myeloid neoplasm (Fig. 7c). Additionally, KEGG enrichment analysis highlighted noteworthy pathways, such as ribosome biogenesis in eukaryotes, nucleocytoplasmic transport, and B cell receptor signaling pathway (Fig. 7d).

### Protein–protein interaction network analysis

**Figure 8:**
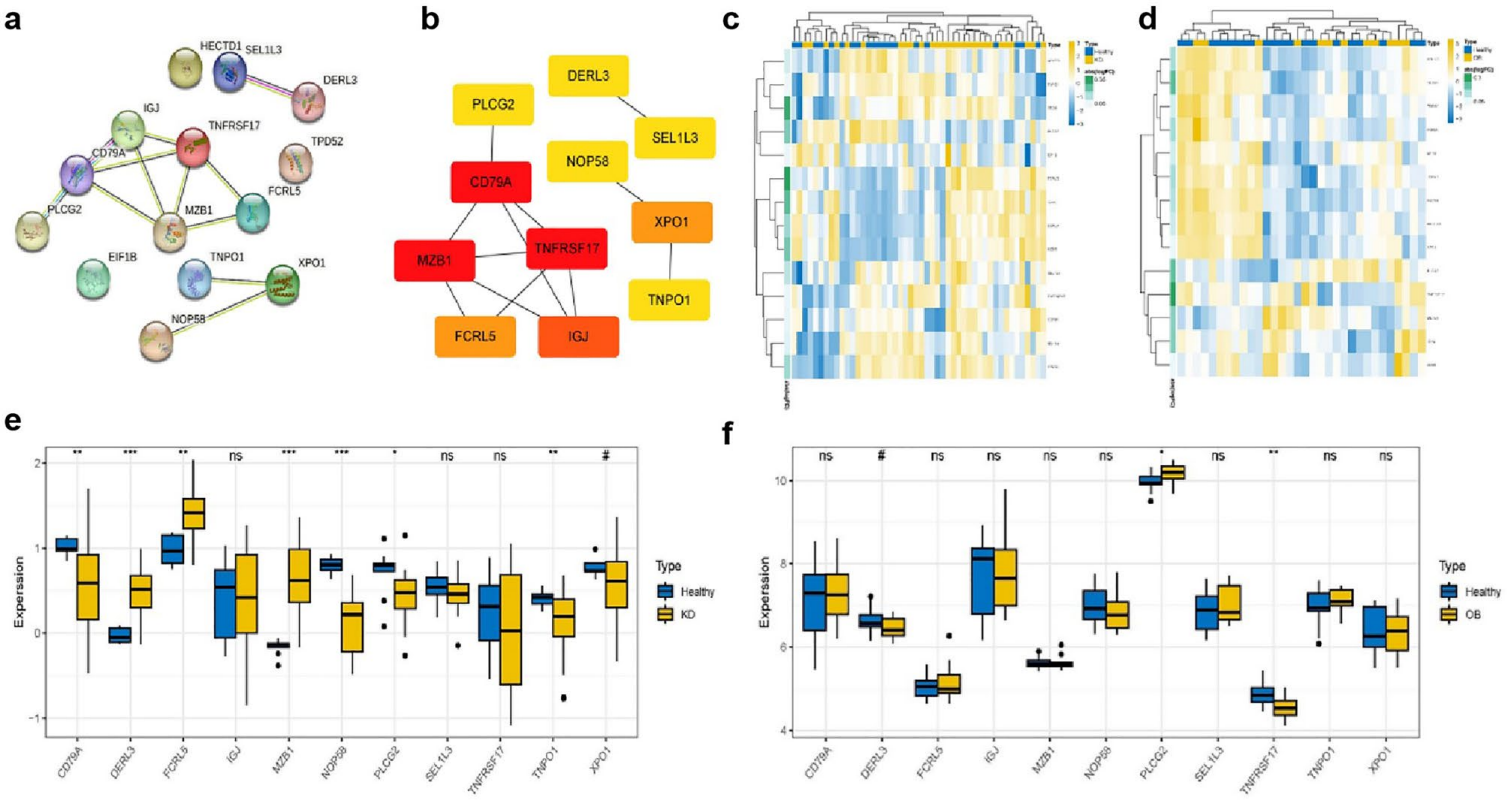
(**a**) Protein–protein interaction (PPI) analysis of the interaction network of 14 key genes, of which 11 genes that are linked are core genes. (**b**) Expression levels of 11 core genes in KD and OB. (**c** and **d**) Heat map of overall expression of core genes in KD and OB patients: healthy control samples in blue, disease samples in yellow, high expression in yellow and low expression in blue. Genes are listed on the horizontal axis, while gene expression levels are shown on the vertical axis. (**e** and **f**) Boxplot showing the total expression of key genes in KD and OB patients. (^#^P < 0.02; *P < 0.05; **P < 0.01; ***P < 0.001.)

The STRING database was used to construct a 14-node PPI network potentially instrumental in B cell genesets of KD and OB (Fig. 8a). Subsequently, visualization of the top highly ranked up-regulated genes was accomplished via Cytoscape software (Fig. 8b). For a comprehensive analysis of overall expression, we presented heatmaps showcasing core gene expression levels in KD, OB, and control samples (Fig. 8c,d). Significantly, the histogram of core gene expression levels brought attention to the substantial expression of DERL3, FCRL5, and MZB1 in KD (Fig. 8e), as well as the heightened expression of PLCG2 in OB (Fig. 8f). To evaluate specificity and sensitivity, ROC curves were generated based on 11 candidate hub genes, ultimately allowing the identification of specific genes associated with the disease (Fig. S9).

### Correlation analysis of core genes

**Figure 9:**
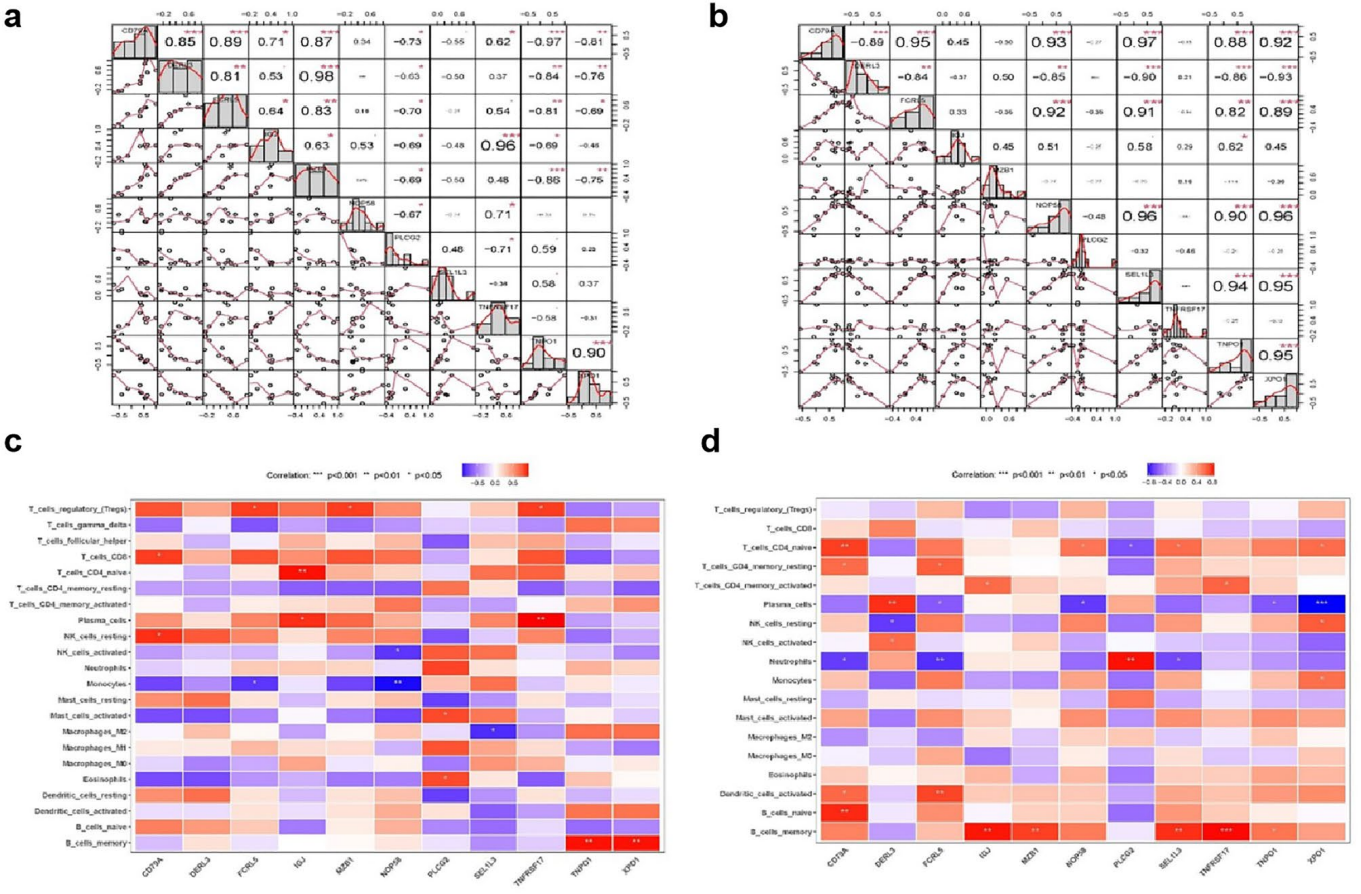
(**a** and **b**) Correlation analysis between 11 core genes and two disease. * represents the significance of correlation, and the number represents the degree of correlation. (**c** and **d**) Correlations between immune cells and 11 core genes (*p < 0.05; **p < 0.01; ***p < 0.001).

Our study involved a comprehensive exploration of the correlation between the 11 core genes and their interactions with immune infiltrating cells. The visualization of these correlations can be found in Fig. 9a,b. Notably, in KD, DERL3 exhibited a correlation coefficient of 0.81 with FCRL5 and an even higher correlation coefficient of 0.98 with MZB1. Additionally, FCRL5 displayed a correlation coefficient of 0.83 with MZB1. On the contrary, in OB, DERL3 demonstrated a negative correlation with FCRL5, while PLCG2 did not show significant correlations with other genes. Furthermore, in KD, T cell CD4 naive displayed a positive correlation with IGJ, and Plasma cells exhibited a positive correlation with TNFRSF17. Moreover, B cell memory showed positive correlations with TNPO1 and XPO1. Similarly, in OB, B cell memory exhibited positive correlations with IGJ, MZB1, SEL1L3, and TNFRSF17 (Fig. 9c,d).

### TNFRSF17 exhibited a causal association with the risk of cardiovascular disease

**Figure 10:**
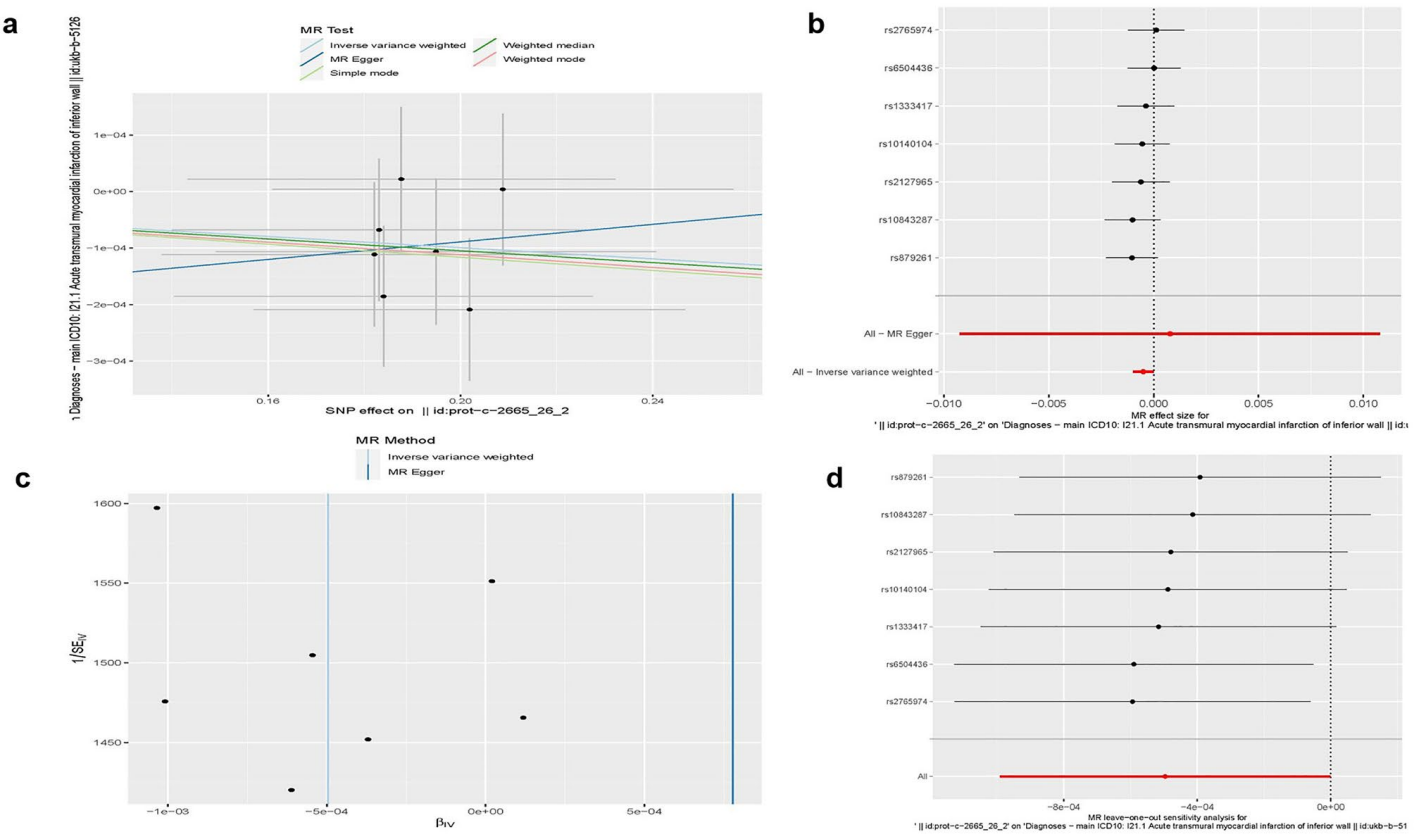
Results of a Mendelian randomization trial. (**a**) Scatter plot illustrating how TNFRSF17 affects myocardial infarction risk causally. (**b**) A forest plot illustrating how each SNP affects the risk of myocardial infarction. (**c**) The impact of TNFRSF17 on myocardial infarction is shown in funnel plots, which show the general heterogeneity of the MR estimations. (**d**) The leave-one-out plot illustrates the causal relationship between TNFRSF17 and the risk of myocardial infarction when one SNP is removed.

Table S1 includes the SNP characteristics of TNFRSF17 and myocardial infarction. It is important to note that all SNPs served as robust instrumental variables, ensuring the accuracy of our analysis. Our investigation centered on examining the causal effects of genetic variations on cardiovascular disease, with special attention to the relationship between TNFRSF17 levels and myocardial infarction (Fig. 10a,b). The IVW method revealed a significant association between TNFRSF17 and myocardial infarction risk, with an odds ratio (OR) of 0.9995 (95% CI = 0.9990–1.0000, p = 0.049). Conversely, the MR–Egger method yielded non-significant results [OR = 1.0008, 95% CI = 0.9908–1.0109, p = 0.885]. Importantly, the funnel plot demonstrated a symmetrical pattern (Fig. 10c), and the MR Egger regression exhibited non-horizontal pleiotropy (p = 0.813), confirming the absence of pleiotropy-induced bias in the causal effect. Moreover, Fig. 10d depicted a systematic MR analysis, excluding each SNP while consistently upholding the significance of causality. This evidence highlights the lack of a dominant SNP affecting TNFRSF17 levels and myocardial infarction, thereby affirming the credibility of our previous MR findings.

### Validation of hub gene expression in KD and OB

**Figure 11:**
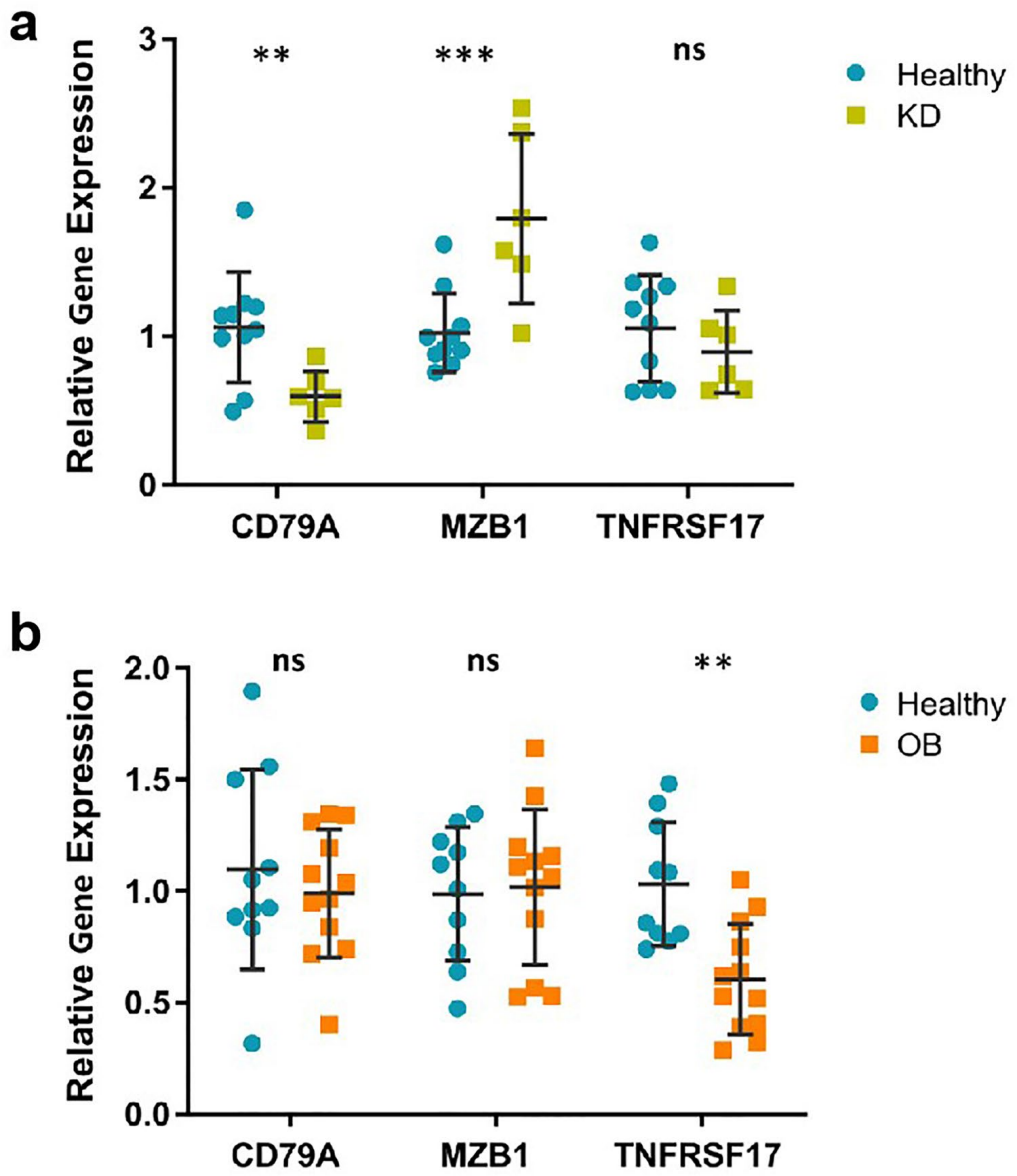
Validation of the differential expression of hub genes, CD79A, MZB1 and TNFRSF17, via qRT‑PCR. *, P < 0.05; **, P < 0.01; ***, P < 0.001; ns, not significant.

qRT-PCR was used to assess the expression levels of CD79A, MZB1, and TNFRSF17 in six KD blood samples and ten control blood samples. A significant difference (P < 0.05) was seen in two genes: When comparing KD samples to control samples, MZB1 was elevated and CD79A was downregulated. There was no discernible change between the control and KD samples according to TNFRSF17 (Fig. 11a). Then, ten control blood samples and three OB blood samples were used to assess the expression levels of CD79A, MZB1, and TNFRSF17. While CD79A and MZB1 did not significantly vary between OB samples and control samples, TNFRSF17 was downregulated in OB samples (Fig. 11b).

## Discussion

Childhood obesity has emerged as a pressing global public health challenge in recent years^3^. Adipocytes are believed to wield the power to activate immune cells, thereby involving themselves passively in inflammatory processes within specific tissues^27,28^. Additionally, adipose tissue contributes to systemic inflammation by synthesizing and secreting various products, among which pro-inflammatory cytokines and chemokines act as chemical attractants and activators of specific immune cells^29,30^. Moreover, adipose tissue serves a dual role, acting both as an inflammation source and a target of the inflammatory process^8,31^. The enigmatic Kawasaki disease presents as systemic vasculitis, prominently characterized by endothelial cell edema and inflammatory cell infiltration. Despite the extensive research, the etiology and pathogenesis of Kawasaki disease remain elusive. Nevertheless, lipid-derived cytokines Omentin-1 and Chemerin have emerged as potential inflammation mediators that might play an important role in the pathogenesis of KD^32^. This study employs an innovative approach by integrating transcripts from Kawasaki disease and obesity, employing single-cell analysis to uncover shared mechanisms between the two conditions. In doing so, the study uncovers potential crosstalk genes, common pathways, and associated immune cells. The implementation of bioinformatics techniques has notably enriched the diversity of disease research and analytical methods^33^.

In this study, we used single-cell analysis for the first time to investigate the immune cell subtypes that interact between KD and OB. Both CIBERSORT and GSEA analyses reveal significant differences in B-cell subpopulation genes between the control group and the groups with KD and obese children. In the Kawasaki disease group, B cell naive levels were notably higher compared to the control group, aligning with prior findings. Moreover, Monocyte and B cell proportions were increased in KD patients before treatment. Studies indicate that during the acute phase of KD, T cells decrease significantly, while B cells increase significantly. Given the prevalence of KD in the Asian population, a series of susceptibility genes may contribute to the high incidence of KD among East Asian individuals. Among the susceptibility genes identified through GWAS, the majority are associated with B cells, contributing significantly to early B cell development and function. This emphasizes the crucial role of B cell immunity in KD development^34^. B cell development takes place in fetal or adult bone marrow, observed in both mice and humans. Conflicting evidence exists on the impact of obesity on bone marrow B cell development, with some studies suggesting enhancement while others suggest impairment^35–37^. In a recent study, C57Bl/6 mice were fed a high-fat diet (HFD) for 180 days, and starting from 90 days after HFD initiation, there was a significant increase in pre-B cells, immature B cells, and mature B cells, persisting throughout the entire study^38^.

After identifying the common immune cell subpopulations between KD and OB, we interacted with the characteristic genes of the immune cell subpopulations in an attempt to identify the interactions between the 11 shared core genes. These core genes are SEL1L3, DERL3, IGJ, CD79A, PLCG2, TNFRSF17, MZB1, FCRL5, TNPO1, XPO1, and NOP58. Using Cytoscape software, we identified TNFRSF17, CD79A, and MZB1 as pivotal genes in the protein–protein interaction (PPI) network and hub gene analysis. The TNFRSF17 gene, a member of the TNF receptor superfamily, also known as B cell maturation antigen (BCMA or BCM) or CD269, plays a role in NF-κB activation, B cell survival, and proliferation^39,40^. A population study in children demonstrated significant correlations between serum sBCMA levels and disease activity, as well as immune and hematology parameters in patients with kidney involvement^41^. And there is research suggesting that TNFRSF17 could serve as a potential therapeutic target for lupus nephritis^42^. CD79A, expressed in B-cell and plasma cell precursors, is a potential candidate for inducing apoptosis and inhibiting B-cell receptor (BCR) activation, which could be relevant to eliminating ectopic germinal centers^43^. In overweight and obese (OW/OB) adults, VAT inflammation and CD79A have been implicated in the development of hypertension^44^. MZB1, a B cell-specific protein that is located in the endoplasmic reticulum, is connected to inflammation and is implicated in a number of inflammatory disorders, including chronic periodontal conditions and several malignancies^45^. According to research, MZB1 is a key regulator of myocardial cells after myocardial infarction and a possible therapeutic target for ischemic cardiomyopathy^46^. It may improve mitochondrial activity and decrease inflammatory signaling pathways. This study aims to explore potential therapeutic targets for reducing the incidence of coronary artery disease (CAD) in obese patients with KD. Considering the common link of CAD development in both KD and OB, this investigation identifies three genes that could play crucial roles in aggravating CAD incidence in obese KD patients. Targeted treatments aimed at these specific genes hold promise in ameliorating the progression of CAD, particularly in obese KD patients. We then assessed the expression levels of these 3 genes by qRT-PCR. The expression levels of TNFRSF17, CD79A, and MZB1 were consistent with our bioinformatics analysis results.

The two-sample MR analysis used in this work, which is based on a large GWAS dataset of TNFRSF17 (exposure) and myocardial infarction (outcome), is the first investigation of the causal relationship between TNFRSF17 levels and coronary artery disease. Research suggests that ischemic heart disease is the most common cardiovascular event among KD survivors^47^, hence we chose myocardial infarction as the outcome measure. The results of the MR study suggested a possible causative relationship between elevated myocardial infarct risk and serum TNFRSF17 levels. Similar to prospective RCTs, MR reduces systemic biases seen in conventional observational research, such as confounding variables and reverse causality. Regression dilution brought on by detection mistakes may be efficiently avoided with the use of very precise genotyping. We intentionally chose people from European populations to reduce the impact of any confounding variables between TNFRSF17 and myocardial infarction, ensuring the validity of our results. We also performed an MR-Egger regression test to further evaluate result stability, although this test did not reveal any directional pleiotropy.

Although our research has yielded meaningful findings, it does have some limitations. First off, this study's investigation of juvenile OB and KD illness used one single-cell dataset and one transcriptome dataset. The scarcity of available single-cell datasets implies that combining more disease datasets would significantly enhance the meaningfulness of our findings. Secondly, to confirm the hub gene expression levels in KD and OB, our investigation was restricted to small sample numbers using qRT-PCR.

## Conclusions

Using single-cell analysis, we identified comorbidity characteristic immune cell subpopulations of OB and KD, enabling the identification of key genes within these immune subpopulations. This approach may aid in the pre-diagnosis of cardiovascular disease stemming from the comorbidity of OB and KD, while also deepening our understanding of the molecular mechanisms of risk genes.

### Supplementary Information

Supplementary Information.

## Data Availability

The datasets (GSE168732, GSE163830, GSE18606, and GSE87493) generated during and/or analyzed during the study are available from the public repository, and all detailed information is recorded in material and methods.
