# Supplementary material for: B-cell hub genes play a cardiovascular pathogenic role of in childhood obesity and Kawasaki disease as revealed by transcriptomics-based analyses
Source: Sci Rep. 2024 Jul 8;14:15671. doi: 10.1038/s41598-024-65865-w (PMC11231228; doi:10.1038/s41598-024-65865-w)

**Figure S3.** Preliminary analysis of single-cell sequencing of KD.(a) Volcano map visualization of highly variable signature genes and tagging of the top ten highly variable genes. (b) The P-value of each PC was visualized using "JackStrawPlot". (c) The number of PCs to be utilized for clustering was decided upon using an elbow plot. (d) Heatmap of the top 20 PCs. (e) UAMP visualization of 27 cell clusters. (f) tSNE visualization of 27 cell clusters. (g) Clustering trees at different resolutions. (h) Heatmap of differential gene expression in 27 cell clusters.


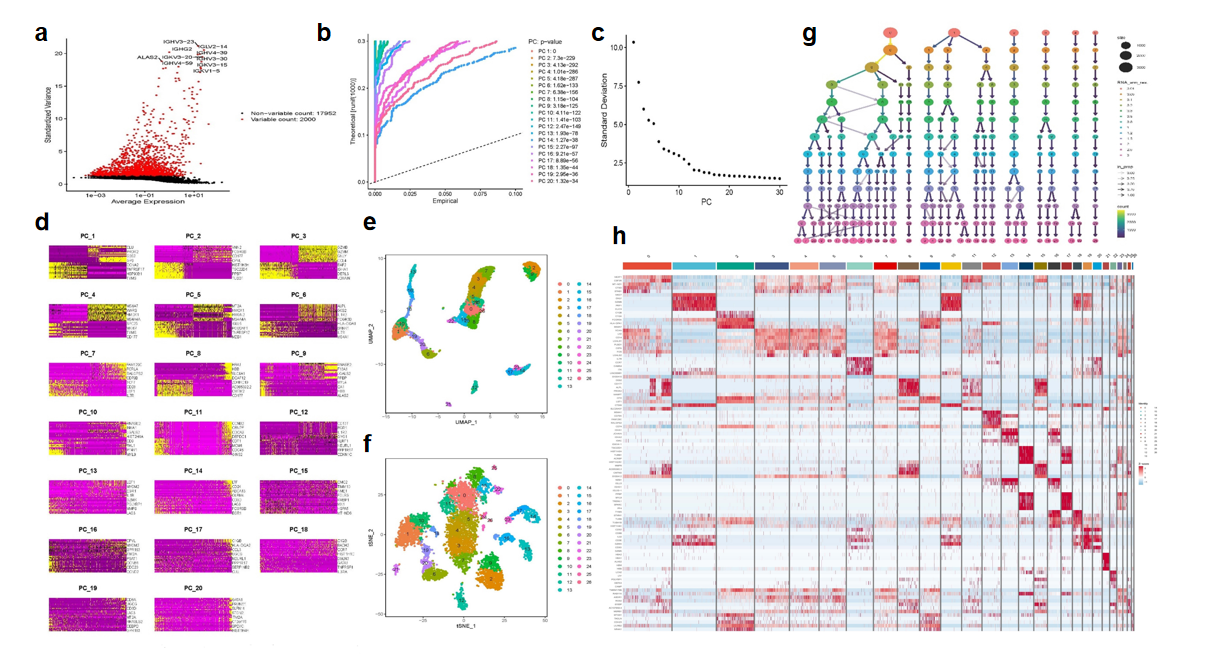

Supplement: Supplementary file 1 — Supplementary Information. [file 41598_2024_65865_MOESM1_ESM.zip › supplementary files/supplymentary figure3.docx]
