# Supplementary material for: B-cell hub genes play a cardiovascular pathogenic role of in childhood obesity and Kawasaki disease as revealed by transcriptomics-based analyses
Source: Sci Rep. 2024 Jul 8;14:15671. doi: 10.1038/s41598-024-65865-w (PMC11231228; doi:10.1038/s41598-024-65865-w)

**Figure S4.** (a) Heatmap of single R annotated. (b) Artificially annotated to 12 cell types. (c) Artificial annotation correction to 10 cell types of tSNE visualization. (d) Artificial annotation correction to 10 cell types of UAMP visualization.


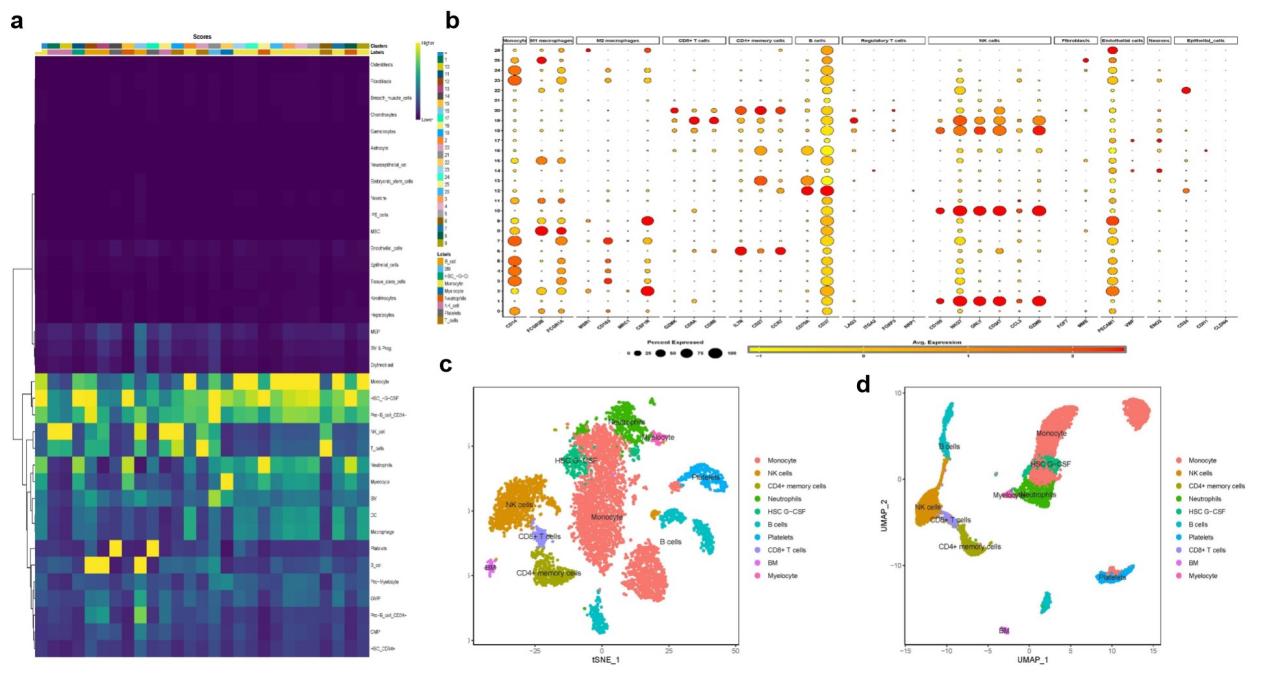

Supplement: Supplementary file 1 — Supplementary Information. [file 41598_2024_65865_MOESM1_ESM.zip › supplementary files/supplymentary figure4.docx]
