# Supplementary material for: B-cell hub genes play a cardiovascular pathogenic role of in childhood obesity and Kawasaki disease as revealed by transcriptomics-based analyses
Source: Sci Rep. 2024 Jul 8;14:15671. doi: 10.1038/s41598-024-65865-w (PMC11231228; doi:10.1038/s41598-024-65865-w)

**Figure S7.** In OB (a) Density of the cell cycle. (b) Distribution of 7 cell types with different cell cycles. (c) Visualizing the cell cycle for tSNE. (d) GSVA results of the HALLMARK pathway for different cell types.


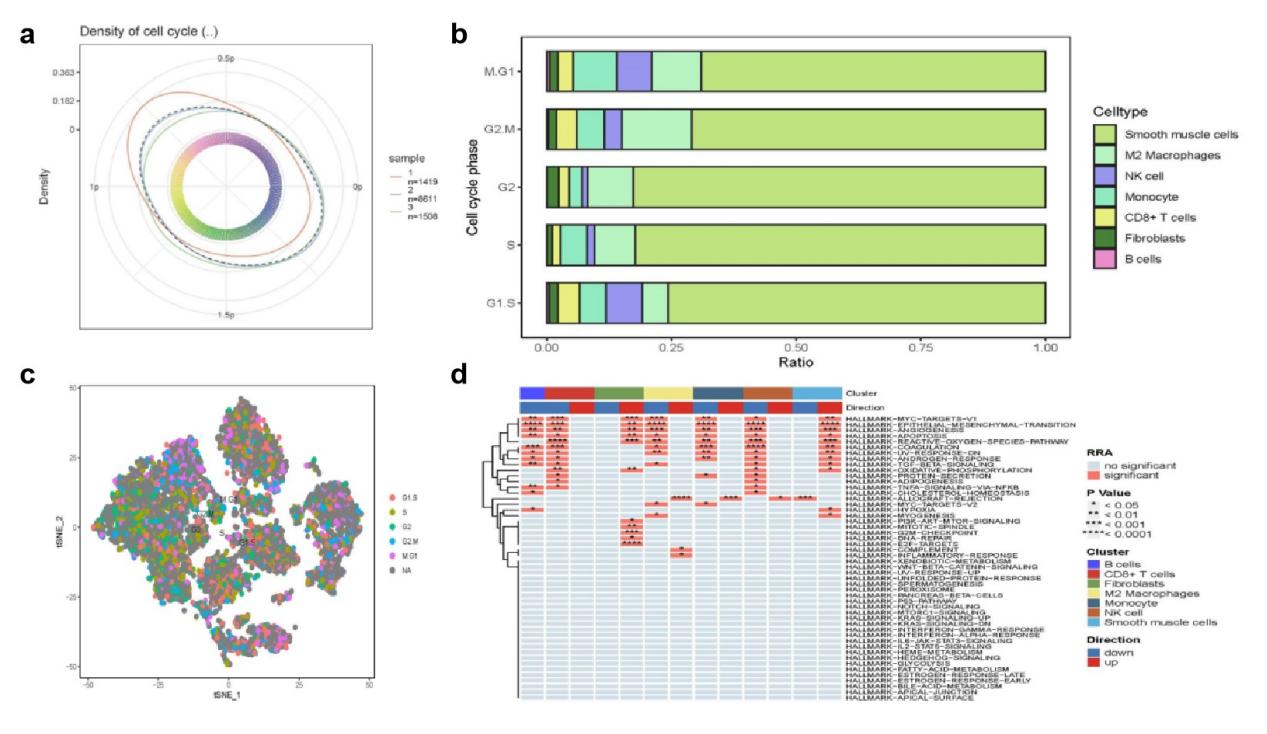

Supplement: Supplementary file 1 — Supplementary Information. [file 41598_2024_65865_MOESM1_ESM.zip › supplementary files/supplymentary figure7.docx]
