# Supplementary material for: B-cell hub genes play a cardiovascular pathogenic role of in childhood obesity and Kawasaki disease as revealed by transcriptomics-based analyses
Source: Sci Rep. 2024 Jul 8;14:15671. doi: 10.1038/s41598-024-65865-w (PMC11231228; doi:10.1038/s41598-024-65865-w)
Supplement: Supplementary file 1 — Supplementary Information. [file 41598_2024_65865_MOESM1_ESM.zip › supplementary files/Table S1.docx]

| Gene Names | Forward （5'-3') | Reverse (3ʹ-5ʹ) |
| --- | --- | --- |
| GAPDH | GGAAGCTTGTCATCAATGGAAATC | TGATGACCCTTTTGGCTCCC |
| TNFRSF17 | GGCTAACATTGACCTGGAAAAGA | TCGAGGCCTCTCGGAAGAA |
| MZB1 | ATGTACTCAGCCCACATGCC | AACTCCGTAGTCCTGCCAGT |
| CD79A | TCTTCCTCCTCTTCCTGCTGTCTG | CGTTGGCGTTGTTGCTGCTATTG |
